# Systematically Displaying the Pathogenesis of Keratoconus *via* Multi-Level Related Gene Enrichment-Based Review

**DOI:** 10.3389/fmed.2021.770138

**Published:** 2022-01-24

**Authors:** Xiao-Dan Hao, Hua Gao, Wen-Hua Xu, Chan Shan, Ying Liu, Zhi-Xia Zhou, Kun Wang, Pei-Feng Li

**Affiliations:** ^1^Institute for Translational Medicine, The Affiliated Hospital of Qingdao University, College of Medicine, Qingdao University, Qingdao, China; ^2^State Key Laboratory Cultivation Base, Shandong Provincial Key Laboratory of Ophthalmology, Shandong Eye Institute, Shandong First Medical University and Shandong Academy of Medical Sciences, Qingdao, China; ^3^Shandong Eye Hospital, Shandong Eye Institute, Shandong First Medical University and Shandong Academy of Medical Sciences, Jinan, China; ^4^Department of Inspection, The Medical Faculty of Qingdao University, Qingdao, China

**Keywords:** keratoconus, candidate genes, multi-level combined analysis, gene enrichment, pathogenesis

## Abstract

Keratoconus (KC) is an etiologically heterogeneous corneal ectatic disorder. To systematically display the pathogenesis of keratoconus (KC), this study reviewed all the reported genes involved in KC, and performed an enrichment analysis of genes identified at the genome, transcription, and protein levels respectively. Combined analysis of multi-level results revealed their shared genes, gene ontology (GO), and pathway terms, to explore the possible pathogenesis of KC. After an initial search, 80 candidate genes, 2,933 transcriptional differential genes, and 947 differential proteins were collected. The candidate genes were significantly enriched in extracellular matrix (ECM) related terms, Wnt signaling pathway and cytokine activities. The enriched GO/pathway terms of transcription and protein levels highlight the importance of ECM, cell adhesion, and inflammatory once again. Combined analysis of multi-levels identified 13 genes, 43 GOs, and 12 pathways. The pathogenic relationships among these overlapping factors maybe as follows. The gene mutations/variants caused insufficient protein dosage or abnormal function, together with environmental stimulation, leading to the related functions and pathways changes in the corneal cells. These included response to the glucocorticoid and reactive oxygen species; regulation of various signaling (P13K-AKT, MAPK and NF-kappaB), apoptosis and aging; upregulation of cytokines and collagen-related enzymes; and downregulation of collagen and other ECM-related proteins. These undoubtedly lead to a reduction of extracellular components and induction of cell apoptosis, resulting in the loosening and thinning of corneal tissue structure. This study, in addition to providing information about the genes involved, also provides an integrated insight into the gene-based etiology and pathogenesis of KC.

## Introduction

Keratoconus (KC) is a complex multifactor degenerative disorder of the cornea, characterized by corneal ectasia, thinning, and cone-shaped protrusion, leading to reduced vision, irregular astigmatism, and corneal scarring (1–4). The worldwide prevalence of KC is approximately 1:2000 (4). KC usually manifests during puberty, and the clinical manifestation vary depending on disease severity (3). Myopia and astigmatism in one or both eyes were the main symptoms in the early stage. With disease progression, visual acuity of patients is progressive loss, and cannot be corrected with spectacles. KC at completion stage often has typical clinical sign, including Munson sign, a V-shape deformation of the lower eyelid in downward position; Fleischer ring, a hemosiderin arc or circle line around the cone base; Vogt's striae, fine vertical lines produced by Descemet's membrane compression (3, 4). In addition, the central or lower temporal part of the cornea shows obvious conical protrusion, and the central cornea becomes thinner obviously. In the completion stage, KC spontaneously or due to external forces such as eye rubbing, rupture of the posterior elastic layer of the cornea occurs, resulting in acute corneal edema and significant decline in visual acuity (3, 4). Because of the unclear pathogenesis and limited availability of medical treatments, KC has become a significant clinical problem worldwide and a leading indication for corneal transplantation (5).

Probing KC's etiology and pathogenesis and adopting effective control methods are the fundamentals of prevention and treatment of KC. KC has a clear genetic tendency. Genetic factors are involved in the development of KC (2, 6, 7). Until now, more than 70 candidate genes and regions have been screened and identified by genome-wide linkage analysis, whole-exome sequencing (WES), candidate gene sequencing, genome-wide association study (GWAS), or candidate gene association study (7–90). Due to the genetic heterogeneity and population differences among KC patients, the genetic cause of most cases has not been effectively identified, and the pathogenesis underlying the genetic mutation remains unclear. This represents the current bottleneck in KC etiology research, so it is very important to find a breakthrough point to explore the key related genes, and the common pathogenesis, of KC.

Traditional genetic studies have typically focused on high-quality families to map and identify new disease-causing genes or screen susceptible sites through population association analysis. However, the pathogenesis caused by mutations or susceptible sites is obscure, leading to the slow progress of pathogenesis studies of most genetic diseases, especially complex ones (91). The combined analysis of multi-levels can achieve the display of the candidate genes screened at the DNA level at the transcription level, the analysis of mutations in the genome at the RNA level for significantly differentially expressed genes, and the enrichment analysis of key genes in the pathway. Integrated genomics, transcription, and protein data can be leveraged to systematically analyze multiple consecutive events occurring in diseases, including changes in expression levels caused by gene mutations, and various forms of heterogeneity in transcriptional regulation, translation, and post-translational regulation body and feedback regulation. According to the changes in candidate factors at different levels, the candidate pathogenic factors can be thoroughly explored and the target of pathogenicity can be identified. Multiomics analysis can also be used to build a gene regulatory network in order to clarify the regulatory and causal relationships between various molecules, so as to gain a deeper understanding of the molecular mechanism and genetic basis of complex traits in genetic diseases.

In this analytical study, we examined all the reported genes involved in KC and performed an enrichment analysis of genes identified at the genome, transcription, and protein levels, respectively. In addition, by using gene set enrichment analysis, we attempted to explore the important mechanisms at different levels. Combined analysis of multi-level results revealed their shared genes, GOs, and pathways, allowing us to explore the possible pathogenesis of KC. The results of this study, in addition to providing information about the changed genes involved in the disease, provide an integrated insight into the common pathogenesis of KC.

## Materials and Methods

### Literature Search to Find Relevant Genes

To find genes associated with KC, the literature was reviewed and data were collected manually. All the studies describing genome changes (including pathogenic mutations or susceptible variants) and differentially expressed encoding genes at the transcription and protein levels were scrutinized using the following keywords in the PubMed and Web of Science databases: “keratoconus” AND (“gene” OR “expression” OR “transcriptome”). We limited our search to articles published up to search date that were written in English. The search was conducted in November 2020. Then, each article was read and classified carefully. For the genome level, only those studies on patients with keratoconus were collected, excluding those on other syndromes patients with keratoconus, central corneal thickness and corneal curvature of normal person unless their results were confirmed in keratoconus samples. For the transcription and protein levels, only those human studies that directly used *in situ* KC corneal tissue or primary KC corneal cells were selected. Finally, we identified and recorded all the reported genes with pathogenic mutations or susceptible variants, differentially expressed genes at the transcription level, and differentially expressed or abnormally distributed genes at the protein level. This study did not search other databases, such as clinical trials and so. The relevant genes collected just included the genes published in the PubMed and Web of Science databases.

### Enrichment Analysis

Enrichment analysis is a statistics-based method for classifying genes that are overrepresented in a specific set of genes. All the genes associated with KC were classified into three groups according to changed levels, and gene ontology (GO) and Kyoto Encyclopedia of Genes and Genomes (KEGG) pathway analysis were performed *via* the online Database for Annotation, Visualization, and Integrated Discovery (DAVID) software, version 6.8 (92, 93).

### Combined Analysis of Multi-Levels

Combined analysis of multi-level enrichment revealed their shared GOs and pathways, allowing us to explore the possible pathogenesis of KC. To further identify the putative pathogenicity of gene mutations/susceptible variants and to detect the KC-related gene function and pathway changes, we conducted a combined analysis of the multi-level results. Online tools (http://bioinformatics.psb.ugent.be/webtools/Venn/) were used to calculate and draw custom Venn diagrams depict the genes, GOs, and KEGG pathways shared by the multi-level results.

## Results

After an extensive review of resources, more than 200 studies were selected from the published articles and were reviewed in greater detail. Our study confirmed the 80 candidate genes, 2,933 differentially expressed genes at the transcription level, and 947 differentially expressed genes at the protein level.

### Candidate Genes and GO/Pathway Enrichment at the Genomic Level

**Figure 1:**
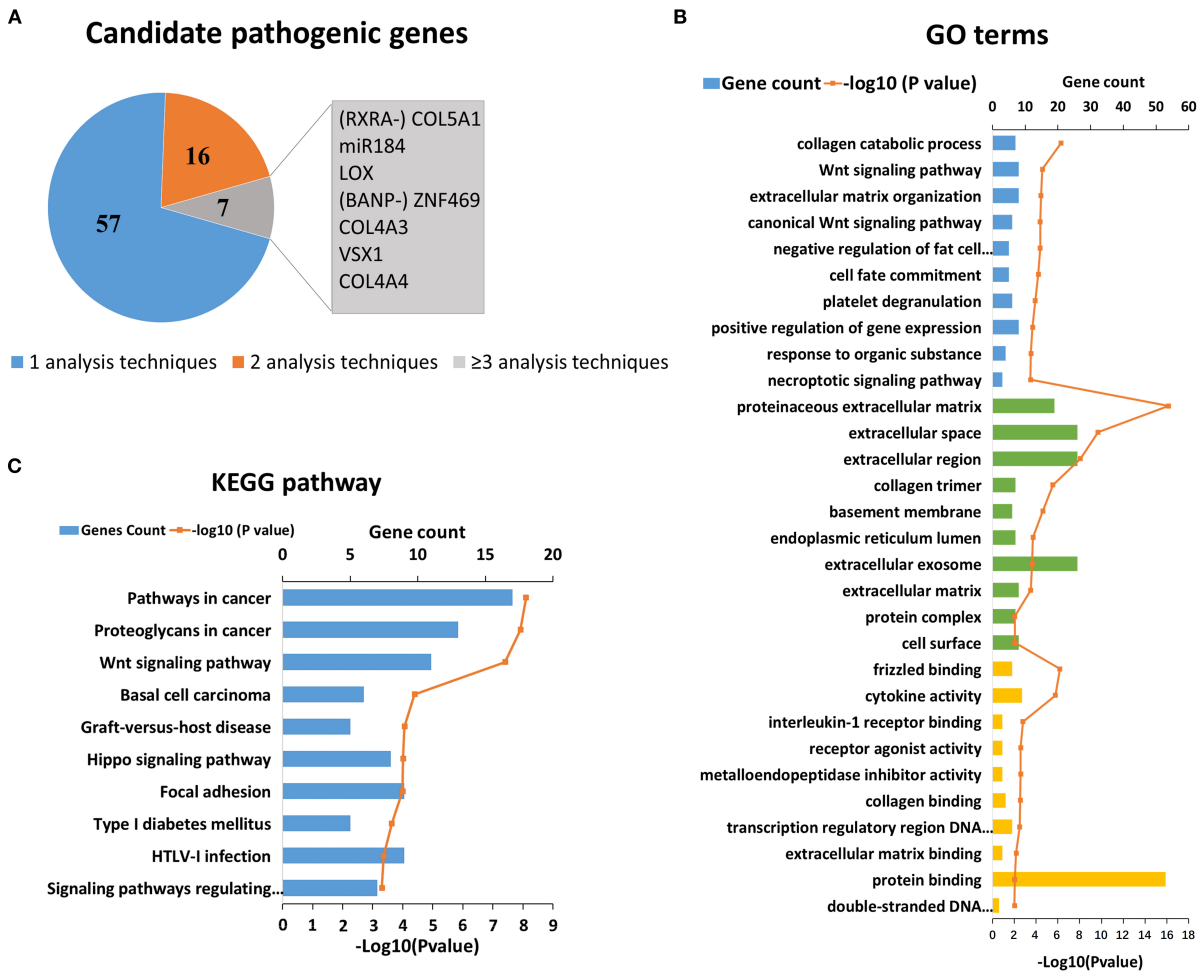
Reported candidate genes in keratoconus at the DNA level. **(A)** The associated genes identified by different analysis techniques. **(B)** Top ten enriched terms of each GO category at the DNA level. The three colors represent biological process (blue), cell component (green), and molecular function (yellow), respectively. **(C)** Top ten enriched KEGG pathways at the DNA level.

KC has a clear genetic tendency (2, 6, 7). In the past 20 years, scholars have extensively investigated the genetic cause of KC (7–90). Thus far, more than 40 candidate genes and regions have been located and screened *via* genome-wide linkage analysis, WES, or candidate gene sequencing. In addition, candidate gene association studies and GWAS have been carried out successively. Thus far, research has found that SNP (Single Nucleotide Polymorphism) loci of 30 genes are related to KC, which can increase or decrease the risk of KC. After an initial search, 80 candidate genes were collected for further analysis. As the results show, a few genes were identified by more than one type of analysis, which represents stronger evidence of their involvement in KC (Figure 1A). The top seven genes were *COL5A1, MIR184, LOX, ZNF469, VSX1, COL4A3* and *COL4A4* (Figure 1A). Detailed literature informations of reported KC associated genes at the genomic level were shown in Supplementary Table S1.

On the basis of the DAVID results, we tested whether the genes associated with KC clustered into certain GO terms and KEGG pathways. Figures 1B,C shows the significant GO terms and KEGG pathways with a *p* < 0.05. We found that the top-ranking GO terms were proteinaceous extracellular matrix (ECM) (GO: 0005578, *p* = 6.00 × 10^−17^), extracellular space (GO: 0005615, *p* = 2.10 × 10^−10^) and their related pathways in the cell component category, including extracellular region, collagen timer, basement membrane, extracellular exosome, as well as ECM (Figure 1B). The top five enriched biological processes were collagen catabolic process (GO: 0010033, *p* = 5.00 × 10^−7^), Wnt signaling pathway (GO: 0009611, *p* = 2.60 × 10^−5^), ECM organization (GO: 0033554, *p* = 3.50 × 10^−5^), canonical Wnt signaling pathway (GO: 0006916, *p* = 4.20 × 10^−5^), and negative regulation of fat cell differentiation (GO: 0010941, *p* = 4.20 × 10^−5^). The top molecular function terms most were involved in or related to various binding (frizzled binding, interleukin-1 receptor binding, collagen binding, transcription regulatory region DNA binding and ECM binding etc.), and activities of cytokine, receptor agonist, and metalloendopeptidase inhibitor.

The top-ranking KEGG pathways were associated with certain complex diseases, including cancer, type 1 diabetes mellitus, and graft-vs.-host disease (Figure 1C). These results suggested that KC might overlap with some of the pathogenesis of these diseases. The others top-ranking KEGG pathways were Wnt signaling pathway, Hippo signaling pathway, and Focal adhesion, which suggest that these pathways may play a role in the pathogenicity of KC.

### Differential Genes and GO/Pathway Enrichment at the Transcription Level

Detailed literature information of differentially expressed encoding genes were shown in Supplementary Table S2 (49, 90, 94–136). In addition to many studies that focused on particular genes or gene families, other studies investigated the transcriptome. Quantitative real-time PCR (qRT-PCR), RNA-seq and microarray were the main methods used in the differential expression studies (49, 90, 94–136). All the significantly differentially expressed encoding genes were collected regardless of whether the different corneal tissue types including corneal tissue, corneal epithelia, corneal stroma and primary stromal fibroblast.

**Figure 2:**
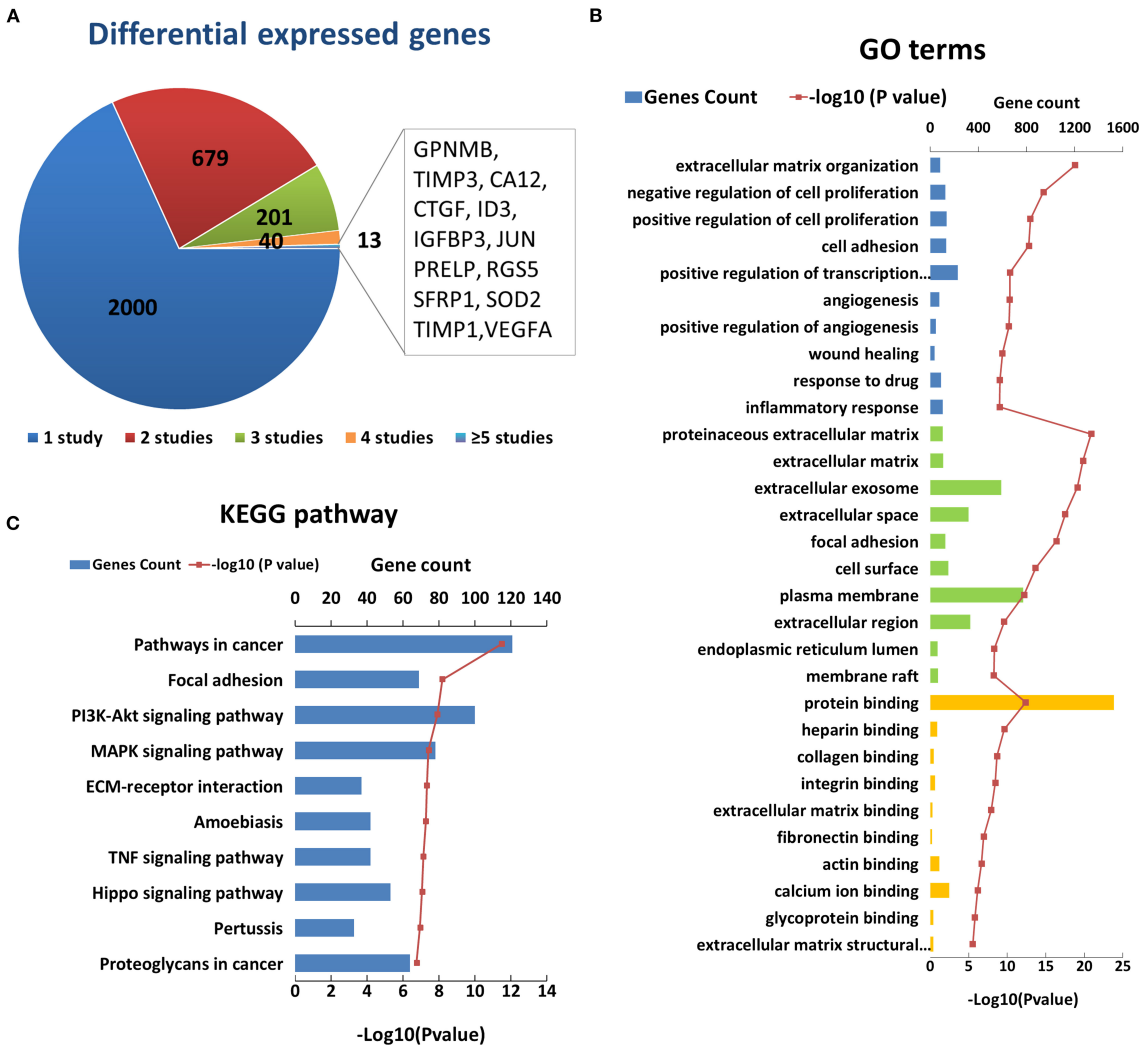
Enriched GO terms and KEGG pathways based on 2933 differential genes at the transcription level. **(A)** The classification of the 2933 differential genes according to the number and results of related studies. **(B)** Top 10 enriched terms of each GO category at the transcription level. The three colors represent biological process (blue), cell component (green), and molecular function (yellow), respectively. **(C)** Top ten enriched KEGG pathways at the transcription level.

After an initial search, a total of 2,933 reported differentially expressed encoding genes between KC corneas and normal corneas at the transcription level were collected (Figure 2A). There are 1,948 downregulated genes, 802 upregulated genes, and 183 genes with opposite results in different studies (Supplementary Table S2). About two-thirds of differentially expressed encoding genes were reported only once in single studies and need to be verified by further research. The remaining 933 genes were reported more than twice in the same or different corneal tissue types (Figure 2A). Among them, 13 genes, including *GPNMB, TIMP3, CA12, CTGF, ID3, IGFBP3, JUN, PRELP, RGS5, SFRP1, SOD2, TIMP1* and *VEGFA* were reported in five or more studies (Figure 2A). All of these genes were reported significantly down regulated in KC corneal tissues, except for a few opposite results in individual studies. The changes in these genes indicate the decrease of growth factor (*CTGF, IGFBP3* and *VEGFA*), transcription factor (*ID3* and *JUN*), superoxide dismutase (*SOD2*), and metalloproteinase inhibitor (*TIMP3* and *TIMP1*) in KC corneas. These results highlight the importance of these changes in the pathogenicity of KC.

The GO analysis of differentially expressed genes showed that there were 497 biological process, 84 cell component, and 127 molecular factor terms with a *p* < 0.05. The top ten significantly enriched biological process terms were involved in or related to ECM organization, regulation of cell proliferation, cell adhesion, angiogenesis, wound healing, response to drug and inflammatory (Figure 2B). The top ten significant cell component terms were involved in or related to proteinaceous ECM, ECM, extracellular exosome, extracellular space, focal adhesion, cell surface, plasma membrane, extracellular region, endoplasmic reticulum lumen, and membrane raft (Figure 2B). The top ten significant enriched molecular function terms were associated with various bindings, such as protein, heparin, collagen, integrin, extracellular matrix, fibronectin, actin, calcium ion, and glycoprotein bindings. These results further highlight the importance of extracellular region and ECM related proteins in the pathogenicity of KC. In addition, cell proliferation, cell adhesion angiogenesis and response to drug and inflammatory may be involved in the process of KC, which requires further study. The KEGG pathway analysis identified 86 enriched pathways. The top five significantly enriched KEGG pathways were pathways in cancer (hsa05200, *p* = 3.20 × 10^−12^), focal adhesion (hsa04510, *p* = 6.50 × 10^−9^), PI3K-Akt signaling pathway (hsa 04210, *p* = 1.20 × 10^−8^), MAPK signaling pathway (hsa04010, *p* = 3.7 × 10^−8^), and ECM-receptor interaction (hsa04512, *p* = 4.60 × 10^−8^) (Figure 2C). These results suggest that these pathways may play a role in the pathogenicity of KC.

### Differential Genes and GO/Pathway Enrichment at the Protein Level

**Figure 3:**
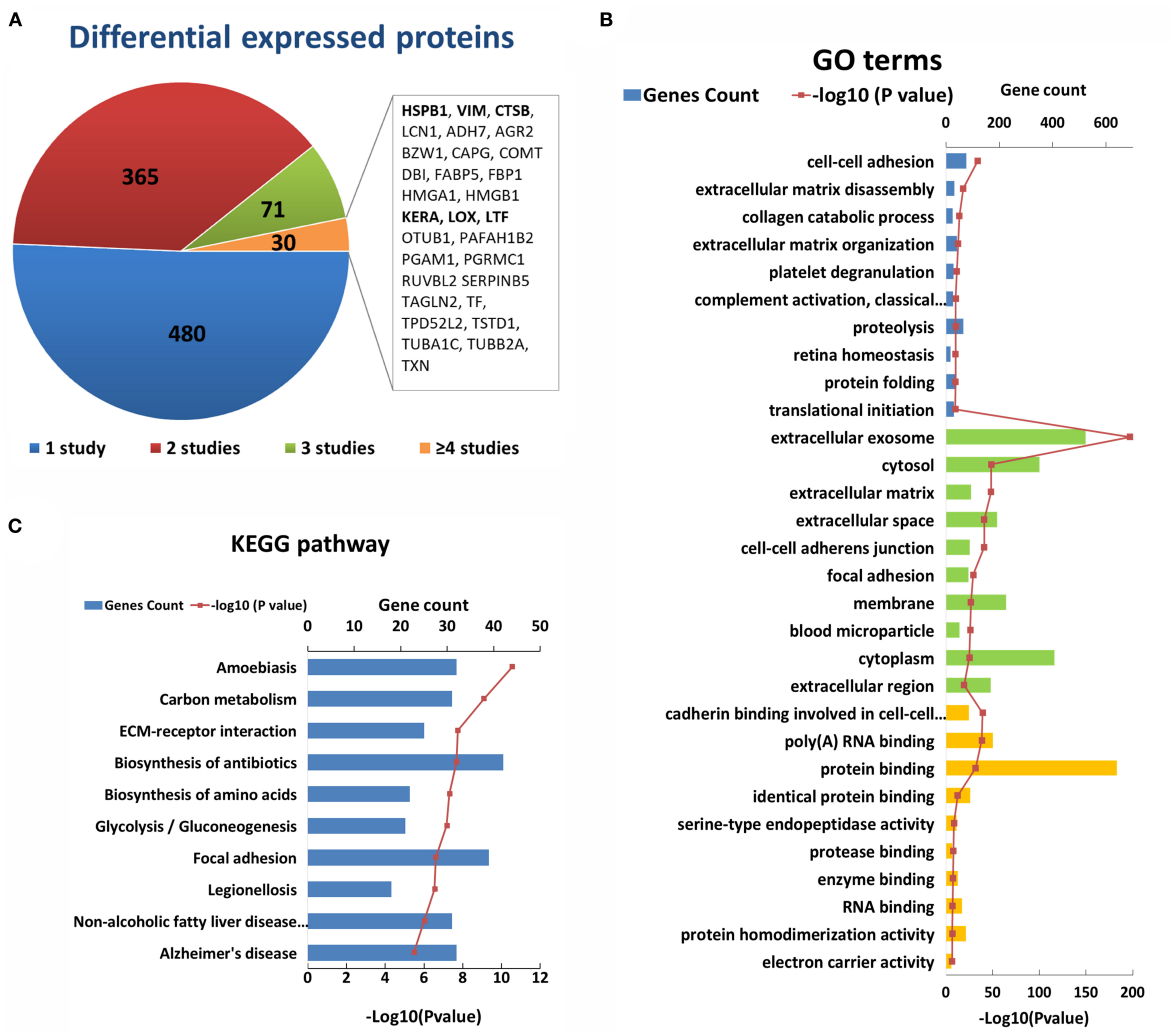
Enriched GO terms and KEGG pathways based on 947 differential genes at the protein level. **(A)** The classification of the 947 differential proteins according to the number and results of related studies. **(B)** Top 10 enriched terms of each GO category at the protein level. The three colors represent biological process (blue), cell component (green), and molecular function (yellow), respectively. **(C)** Top ten enriched KEGG pathways at the protein level.

After an initial search, a total of 946 reported differentially expressed proteins between KC corneas and normal corneas were collected (98–103, 109, 114, 117, 118, 120, 125, 126, 128, 130, 131, 133, 137–193). Immunohistochemistry, immunofluorescence, and western blot were used for candidate protein level detection, and proteomic analysis was performed *via* mass spectrometry. All the reported differentially expressed proteins were collected, including downregulated, upregulated, and abnormally distributed proteins. There are 427 downregulated genes, 398 upregulated genes, 14 abnormally distributed proteins, and 107 genes with opposite results in different studies (Supplementary Table S3). About half differentially expressed genes were reported only once. The remaining 466 genes were reported more than twice in the same or different corneal tissue types (Figure 3A). Among them, 30 genes were reported in four or more times, including five genes (*HSPB1, VIM, HMGA1, TSTD1, RUVBL2*) with upregulation and two genes (*BZW1, LOX*) with downregulation in at least four studies. The changes in these genes indicate the decrease of transcription factor (*BZW1*), lysyl oxidase (*LOX*), and increase of small heat shock protein (*HSPB1*), vimentins (*VIM*), thiosulfate sulfurtransferase (*TSTD1*) and ATPase (*RUVBL2*) in KC corneas. These results highlight the importance of these changes in the pathogenicity of KC. The other genes with conflicting results suggest that the pathogenesis of KC is very complex, and further research is needed to clarify the role of these genes.

The GO analysis of differentially expressed proteins showed that there were 344 biological process, 127 cell component, and 98 molecular factor terms with a *p* < 0.05. The top ten significant enriched biological process terms were involved in or related to ECM disassembly, ECM organization, collagen catabolic process, proteolysis, cell-cell adhesion, platelet degranulation, complement activation, retina homeostasis, protein folding and translational initiation (Figure 3B). The top ten significant cell component terms were involved in or related to extracellular exosome, ECM, extracellular space, extracellular region, cell-cell adherens junction, focal adhesion, cytosol, membrane, cytoplasm, and blood microparticle (Figure 3B). The top 10 significant enriched molecular function terms were associated various bindings (cadherin, RNA, protein, identical protein, protease and enzyme bindings), endopeptidase activity, protein homodimerization activity and electron carrier activity (Figure 3B). These results once again highlight the importance of collagen and ECM in the pathogenicity of KC at the protein level. Except for ECM and its related GO terms, the results showed that proteolysis, cell-cell adhesion, focal adhesion, protein binding, and endopeptidase may be involved in the process of KC (Figure 3B). The top five significant enriched KEGG pathways were Amoebiasis, Carbon metabolism, ECM-receptor interaction, Biosynthesis of antibiotics and Biosynthesis of amino acids (Figure 3C). These results provided further evidence of the important role of ECM pathways in the pathogenicity of KC at the protein level.

### Combined Analysis of Multi-Levels

**Figure 4:**
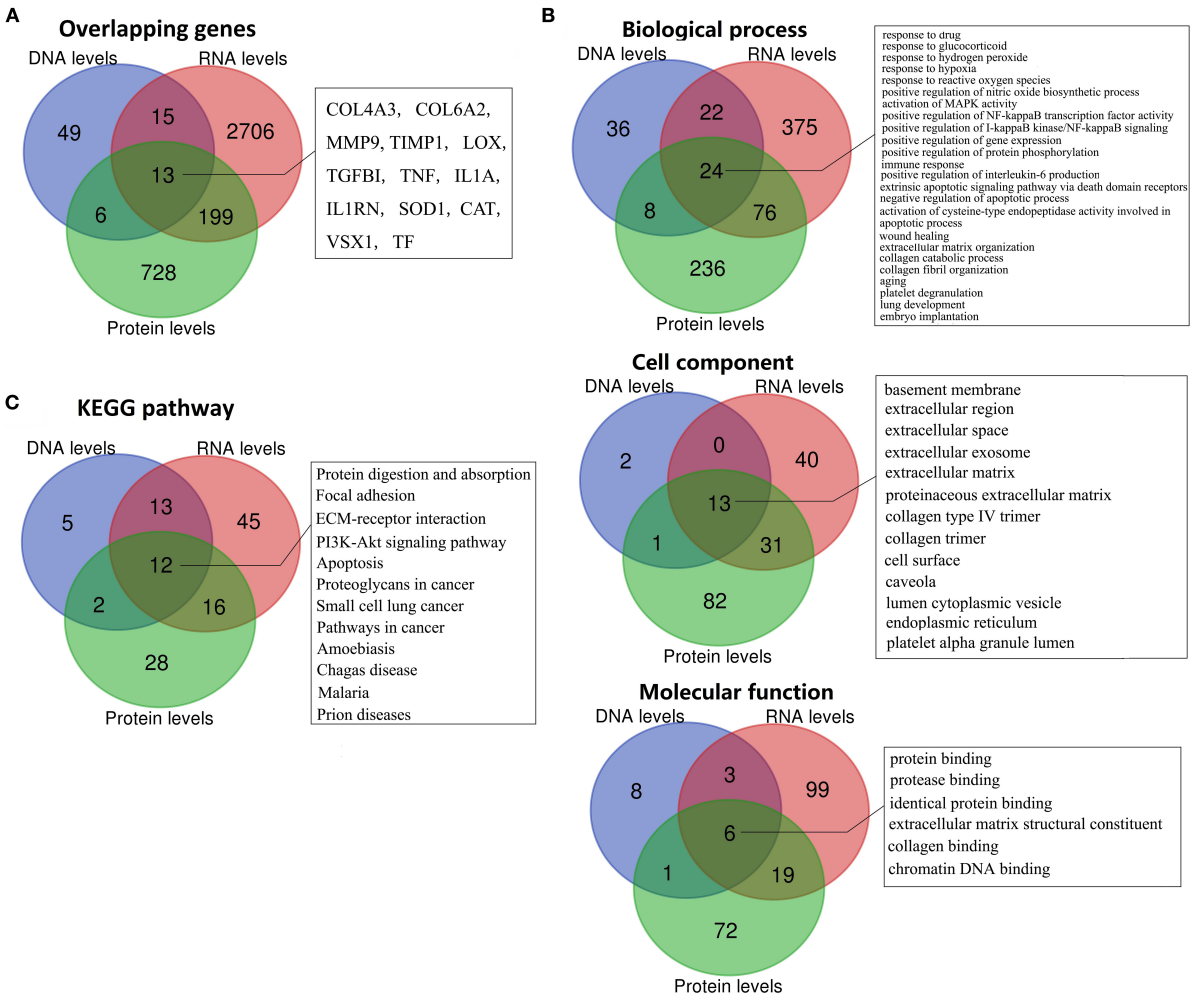
Overlapping genes, GO terms, and KEGG pathways between multi-levels. **(A)** Overlapping genes; **(B)** Overlapping GO terms; **(C)** Overlapping KEGG pathways.

To further identify the putative pathogenicity of gene variants and detect the KC-related gene function and pathway changes, we conducted a combined analysis of DNA, RNA, and protein level results. First, we analyzed the genes shared between the different levels. The results showed that there were 13 overlapping genes between all three levels (Figure 4A, Table 1). They were *COL4A3, COL6A2, MMP9, TIMP1, LOX, TGFBI, TNF, IL1A, IL1RN, SOD1, CAT, VSX1* and *TF*. All the genes except *TGFBI* and *SOD1* were reported to have KC-susceptibility SNPs. Potential pathogenic mutations of seven overlapping genes (*LOX, IL1RN, COL4A3, VSX1, TGFBI, SOD1* and *COL6A2*) were identified in KC patients. In addition, *LOX* and *IL1RN* were located in the susceptible loci detected by linkage analysis. Five of the 13 genes (*LOX, IL1RN, VSX1, COL4A3*, and *TGFBI*) were identified by multiple types of analysis at the DNA level. Three of the 13 genes (*LOX, TIMP1*, and *TNF*) were verified in multiple studies with consistent expression changes at the transcription level. Five of the 13 proteins (*LOX, IL1RN, COL6A2, MMP9*, and *TNF*) were verified in multiple studies with consistent changes at the protein level. These results suggested that these overlapping genes might be key genes of KC and might play an important role in the pathogenesis of KC. As the results show (Table 1), five genes (*TNF, MMP9, IL1A, CAT*, and *VSX1*) had significant upregulation, and six genes (*TIMP1, COL6A2, SOD1, TGFBI, COL4A3*, and *LOX*) had significant downregulation in KC at both the transcription and protein levels. The coincident changes of these genes also indicated the decrease of collagen (*COL4A3* and *COL6A2*), metallopeptidase inhibitor (*TIMP1*), lysyl oxidase (*LOX*), superoxide dismutase (*SOD1*), and the increase of metallopeptidase (*MMP9*), antioxidant enzyme (*CAT*) and inflammatory cytokines (*TNF* and *IL1A*). These results highlight the importance of these changes in the pathogenicity of KC.

**Table 1:** Overlapping genes between multi-levels.

**Genes**	**Analysis techniques of DNA level**	**Changes of RNA level (N)**	**Changes of protein level (N)**
***LOX***	**Linkage analysis; candidate gene mutation analysis; GWAS; candidate gene association studies**	**down (4)**	**down (4)**
*IL1RN*	Linkage analysis; candidate gene association studies	down (1)	up (2)
***COL4A3***	**Candidate gene mutation analysis; NGS; GWAS; candidate gene association studies**	**down (2**)/up (1)	**down (1)**
***VSX1***	**Candidate gene mutation analysis; NGS; candidate gene association studies**	**up (1)**	**up (1)**
***TGFBI***	**Candidate gene mutation analysis; NGS**	**down (1)**	**down (2)**/up (1)
***SOD1***	**Candidate gene mutation analysis**	**down (1)**	**down (1)**/up (2)
***COL6A2***	**NGS**	**down (1)**/up (1)	**down (3)**
***CAT***	**Candidate gene association studies**	**up (1)**	**up (1)**
***IL1A***	**Candidate gene association studies**	down (3)/**up (1)**	**up (1)**
***MMP9***	**Candidate gene association studies**	down (1)**/up (3)**	**up (2)**
*TF*	Candidate gene association studies	down (1)/up (1)	down (1)/up (3)
***TIMP1***	**Candidate gene association studies**	**down (5)**	**down (1)**/up (1)
***TNF***	**Candidate gene association studies**	**up (3)**	**up (3)**

*GWAS, Genome-Wide Association Studies; NGS, Next-Generation Sequencing; N, the number of studies*.

*The genes with consistent changes in transcription and protein levels are bold*.

Combined analysis of different levels' enrichment revealed their shared significant GOs and pathways, allowing the researchers to explore the possible pathogenesis of KC. The overlapping significant GOs between all three levels, including 24 biological process, 13 cell component, and six molecular function terms, are shown in Figure 4B. The shared biological process terms can divided into four groups, including various response-related GOs (response to drug, glucocorticoid, hydrogen peroxide, reactive oxygen species, and hypoxia), apoptosis-related GOs (activation of cysteine-type endopeptidase activity involved in apoptotic process, negative regulation of apoptotic process, and extrinsic apoptotic signaling pathway *via* death domain receptors), ECM-related GOs (ECM organization, collagen catabolic process, and collagen fibril organization) and activation and positive regulation of various signaling (MAPK activity, NF-kappaB activity, and I-kappaB kinase/NF-kappaB signaling). For the cell components, most of the overlapping GOs were ECM related (including basement membrane, ECM, proteinaceous ECM, collagen type IV trimer, and collagen trimer), extracellular related (extracellular region, extracellular space and extracellular exosome) and pinocytosis related (caveola and cytoplasmic vesicle). For the molecular functions, the overlapping GOs were ECM related (ECM structural constituent and collagen binding), and various binding-related GOs (protein, identical protein, protease and chromatin DNA binding). The combined analysis of the KEGG pathway showed that Protein digestion and absorption, Focal adhesion, ECM-receptor interaction, PI3K-Akt signaling pathway, apoptosis, and various diseases related pathways (including cancer, Prion diseases, Malaria, Amoebiasis and Chagas disease) were significantly enriched at DNA, RNA, and protein levels (Figure 4C). Most GO and pathway enrichments shared at DNA, RNA, and protein levels were related to collagen, ECM, extracellular, various responses and apoptosis, suggesting that these GO and pathway changes might have been etiological—serving as mechanisms of KC.

## Discussion

KC is an etiologically heterogeneous corneal ectatic disorder, and both environmental and genetic factors play a role in its etiopathogenesis (194). Based on results from studies that have investigated the genetic etiology, expression, and translation changes in the process of development, it is becoming increasingly clear that KC is a complex disease with a complex etiology or convergence of multiple disease pathways. However, the common pathogenesis underlying the different etiologies remains unclear. In this study, we reviewed all the studies of KC-related genes identified at the genome, transcription, and protein levels. Through multi-level related gene enrichment-based review, we systematically explored the schematic representing factors responsible for KC at different levels. The results of this study, in addition to providing information about the genes involved in the disease, clearly provide an integrated insight into the gene-based etiology and pathogenesis of KC.

Genetic changes play an important role in the etiopathogenesis of KC (2, 6, 7, 194). Many forms of gene variation, such as inheritance gene mutation, *de novo* mutation, and polymorphism, have been reported to be involved in the etiology of KC (7–90). More than half of the genes were reported in one type of study or in single studies. A few genes, encoded chains of collagens (*COL5A1, COL4A3*, and *COL4A4*) (57, 59, 61, 67, 72, 76, 78, 84, 87, 88, 90), collagen cross-linking enzyme (*LOX*) (18, 30, 90), factor for the synthesis or organization of collagen fibers (*ZNF469*) (27, 32, 34, 46, 47, 62, 65), and others (*MIR184* and *VSX1*) (6, 7, 16, 19, 24, 40, 56, 82, 85) were identified in different types of studies, such as pathogenic mutation analysis, polymorphism association analysis, and family-based linkage analysis. However, the occurrence rate of these gene mutations in the population was relatively low, and in many populations, it could not even be verified (25, 195–202), which suggested that KC is genetically heterogeneous. Among the reported KC associated genetic changes, there were 11 genes responsible for apoptosis related process (*FAS, FASLG, TNF, BIRC2, SMAD3, WNT5A, CAT, TIMP1, MMP9, FOXO1*, and *COL4A3*), 14 reported corneal biomechanics loci (*MPDZ, COL6A2, MYOF, LOX, ZNF469, SMAD3, NFIB, FNDC3B, COL5A1, WNT10A, TGFBI, SLC4A11, FOXO1* and *COL4A3*) (203), and five genes responsible for inflammatory processes (*IL1A, IL1B, FAS, TNF* and *IL17B*). Genetic changes in these genes might lead to the changes in related functions and pathways in the corneal cells, then lead to induction of apoptosis, inflammatory and altered biomechanics of cornea, which have been reported involved in the etiopathogenesis of KC (204–206), and lead to the occurrence of KC. The top candidate gene based enrichment, including ECM and their related pathways (76, 90), Wnt signaling pathway (60), and cytokine activities (204), have been reported involved in the etiopathogenesis of KC. The role of negative regulation of fat cell differentiation in the etiopathogenesis of KC has not been reported, but body mass index was reported associated with keratoconus before (207). So, more studies are needed on the relationship between this functional change in order to clarify its relationship and mechanism of action in KC.

Differences between the expression of genes in normal and KC corneas suggested disease pathways. The top 13 verified differentially expressed genes at RNA level indicated a decrease of growth factor, transcription factor, superoxide dismutase, and metalloproteinase inhibitor, which highlights the importance of these changes in the pathogenicity of KC. The top differentially expressed genes based enrichment were similar to the GOs at the genomic level, mainly including ECM and its related GO terms, response to inflammatory and various bindings. These results represent further confirmation of the importance of collagen (61, 67, 75, 78, 84, 87, 88, 113, 117, 125, 163–165), ECM (76, 90, 97, 158), cell adhesion (72, 97), and inflammatory (49, 101, 124, 128, 139, 204) in the pathogenicity of KC. In addition, cell proliferation, angiogenesis, and response to drug have not been reported before, which may be involved in the process of KC, and should be investigated further. For reported differentially expressed proteins, the genes with upregulation or downregulation in at least four studies highlight the importance of transcription factor (98, 106, 112, 128, 145, 160), lysyl oxidase (18, 30, 81, 125, 150, 151), small heat shock protein (156, 188, 193), vimentins (126, 156, 188, 193), thiosulfate sulfurtransferase and ATPase (188) in KC corneas. Gene-based enrichment analysis showed that the differentially expressed proteins were significantly enriched in ECM and its related GO terms, proteolysis, and various bindings. These results once again highlight the importance of these GO terms in the pathogenicity of KC at the protein level.

Different approaches have been used to investigate and define the phenotype, mechanisms, and causes of KC. Thousands of genes were identified at genomic, transcription, and protein levels. Observations of corneal changes that occur in KC often do not distinguish between primary changes and secondary inflammatory or degenerative effects. Although research has identified many differences that distinguish KC corneas from normal corneas, it has not been possible to trace these changes back to primary causes or to identify the triggers that precipitate the cascade of events that leads to the clinical picture of KC. The results at different levels were clearly similar. In order to explore the key points, combined analysis of multi-levels was performed. Integrated genomics, transcription, and protein data can be leveraged to systematically analyze multiple consecutive events occurring in diseases. According to the changes in candidate factors at different levels, the candidate pathogenic factors can be thoroughly explored and the target of pathogenicity can be identified. The consistent changes in these factors at different levels suggested that these factors play an important role in the pathogenesis of KC. The DNA, RNA, and protein changes represented the cause and process changes of KC, respectively.

Based on the results of multi-level combined analysis, we hypothesized that the pathogenic relationships among these related factors is as follows. The etiology of KC can be divided into environmental and genetic factors. The environmental factors may include endogenous and exogenous factors, such as glucocorticoid, hydrogen peroxide, and reactive oxygen species (ROS) (120). The gene mutations or variants involved in collagen (57, 61, 64, 67, 75, 76, 78, 88), metallopeptidase inhibitor (75, 81, 109, 157), lysyl oxidase (18, 30, 81, 90), metallopeptidase (64), antioxidant enzyme (16, 25, 81, 199), inflammatory cytokines (11, 45, 83, 201), and others cause insufficient protein dosage or abnormal function. These genetic changes, together with the aforementioned stimulation, lead to the changes in related functions and pathways in the corneal cells. The related functions include the response to the stimulation of hormones and reactive oxygen species (96, 106, 120, 121, 189), activation and positive regulation of various signaling (MAPK activity, NF-kappaB activity, and I-kappaB kinase/NF-kappaB signaling) (123, 128), upregulation of cytokines and collagen-related enzymes (49, 101, 122, 124, 147, 174, 187), and downregulation of collagen, collagen-crossing, and other ECM-related proteins (97, 103, 117, 163, 164), and regulation of apoptosis (36, 175, 186). These undoubtedly lead to the reduction of extracellular components and the induction of apoptosis and aging. The change in extracellular structure, decrease of extracellular composition, and apoptosis of corneal cells all lead to the loosening and thinning of corneal tissue structure, which leads to the occurrence of KC.

In addition to the different levels and combined analysis results of this paper, our hypothesis was supported by many other studies of molecular mechanisms and cell events of KC. A few hormones and substances have been reported to be associated with KC (208–214). However, the relationship between glucocorticoid and KC has not been studied before, and should be investigated further. Chronic inflammatory events were detected in the tears of KC patients (215–217). A significant increase in the apoptosis of KC cells has been reported in several studies (186, 218, 219). Moreover, a decrease of dulfated epitopes of keratan sulfate KC corneas was also reported (220). The electron microscope results of KC showed that the content of the stroma increases, whereas the fibril diameter is reduced, the mean diameter and interfibrillar spacing of collagen fibrils are reduced, and the collagen fibrils and proteoglycans number density and area fractions are significantly increased (138).

Our study has some limitations. All the results were obtained through multi-level related gene enrichment-based analysis. More studies are needed on the relationship between these functional changes in order to clarify their relationship and mechanism of action, which could provide a new direction for the treatment of KC. For the expression studies collected in this study, there were several different corneal tissue types. Most of the studies used the corneal tissue, and a few studies used the corneal epithelia, corneal stroma or primary stromal fibroblast (see Supplementary Tables S1–S3). In this analysis-based study, because of the limited space, instead of categorizing different genes detected in different tissues, we just conducted a unified analysis in different levels. Analysis that is more detailed needs to be carried out in the future to find the role of different corneal cells in KC pathogenesis. Furthermore, the interaction between genetic factors and environmental factors in the pathogenesis of KC has not been effectively solved, and further research is needed. Epigenetic mechanisms might help explain environmental contributions to the pathogenesis of KC (221). There are few studies on the relationship between epigenetic changes and KC (60). Recently, certain epigenetic changes, such as circle RNA, have been confirmed to play an important role in other diseases having overlapped pathogenesis pathway with KC (222–225), suggesting its potential role in KC pathogenesis. Study the role of these epigenetic changes might be a new research direction of KC in future.

### Conclusions

Keratoconus is an etiologically heterogeneous corneal ectatic disorder, and both environmental and genetic factors play a role in its etiopathogenesis. Based on results from studies that have investigated the genetic etiology, expression, and translation changes in the process of development, it is becoming increasingly clear that KC is a complex disease with a complex etiology or convergence of multiple disease pathways. The common pathogenesis underlying the different etiologies remains unclear. In this study, we reviewed all the studies of KC-related genes identified at the genome, transcription, and protein levels. Through multi-level related gene enrichment-based review, we systematically explored the schematic representing factors responsible for KC at different levels. The results of this study, in addition to providing information about the genes involved in the disease, clearly provide an integrated insight into the gene-based etiology and pathogenesis of KC. Base on the results, we hypothesized that the pathogenic relationships among these related factors is as follows. The gene mutations/variants caused insufficient protein dosage or abnormal function, together with environmental stimulation, leading to the changes in the related functions and pathways in the corneal cells. These included response to the glucocorticoid and reactive oxygen species; regulation of various signaling (P13K-AKT, MAPK and NF-kappaB), apoptosis and aging; upregulation of cytokines and collagen-related enzymes; and downregulation of collagen and other ECM-related proteins. These undoubtedly lead to a reduction of extracellular components and induction of cell apoptosis, resulting in the loosening and thinning of corneal tissue structure. This hypothesis was supported by many other studies of molecular mechanisms and cell events of KC. More studies are needed on the relationship between these functional changes in order to clarify their relationship and mechanism of action, which could provide a new direction for the treatment of KC.

## Data Availability Statement

The original contributions presented in the study are included in the article/Supplementary Material, further inquiries can be directed to the corresponding authors.

## Author Contributions

X-DH and HG designed the research. W-HX, CS, YL, and Z-XZ performed the literature search. X-DH analyzed the data, participated in the discussion, and wrote and revised the paper. KW and P-FL participated in the revision of the paper.

## Funding

This study was supported by National Natural Science Foundation of China (81500763 and 91849209), Shandong Provincial Natural Science Foundation, China (ZR2020MC059), China Postdoctoral Science Foundation (2019M652311), Special Support for Post-doc Creative Funding in Shandong Province, and Applied Research Program for Post-Doctoral in Qingdao.

## Conflict of Interest

The authors declare that the research was conducted in the absence of any commercial or financial relationships that could be construed as a potential conflict of interest.

## Publisher's Note

All claims expressed in this article are solely those of the authors and do not necessarily represent those of their affiliated organizations, or those of the publisher, the editors and the reviewers. Any product that may be evaluated in this article, or claim that may be made by its manufacturer, is not guaranteed or endorsed by the publisher.

## Supplementary Material

Supplementary Table S1 Reported candidate genes in keratoconus at the DNA level.

Supplementary Table S2 The gene lists of reported differential expressed genes between KC and normal cornea at the transcription level.

Supplementary Table S3 The gene lists of reported differential expressed genes between KC and normal cornea at the protein level.
